# Surgical Wound Fluids from Patients with Breast Cancer Reveal Similarities in the Biological Response Induced by Intraoperative Radiation Therapy and the Radiation-Induced Bystander Effect—Transcriptomic Approach

**DOI:** 10.3390/ijms21031159

**Published:** 2020-02-10

**Authors:** Katarzyna Kulcenty, Igor Piotrowski, Marcin Rucinski, Joanna Patrycja Wroblewska, Karol Jopek, Dawid Murawa, Wiktoria Maria Suchorska

**Affiliations:** 1 Radiobiology Laboratory, Greater Poland Cancer Centre, Garbary 15 Street, 61-866 Poznań, Poland; igor.piotrowski@wco.pl (I.P.), wiktoria.suchorska@wco.pl (W.M.S.); 2 Department of Electroradiology, Poznań University of Medical Sciences, Garbary 15 Street, 61-866 Poznań, Poland; 3 Department of Histology and Embryology, Poznan University of Medical Sciences, 60-781 Poznan, Poland; marcinruc@ump.edu.pl (M.R.); kjopek@ump.edu.pl (K.J.); 4 Department of Pathology, Poznan University Medical Sciences and Greater Poland Cancer Center, Garbary 15 Street, 61-866 Poznań, Poland; Joanna.wroblewska@wco.pl; 5 Department of Surgery and Oncology, Faculty of Medicine and Health Sciences, University of Zielona Góra, Licealna 9/9, 65-417 Zielona Góra, Poland, dmurawa@gmail.com; 6 Department of Breast Cancer Surgery, Greater Poland Cancer Centre, Garbary 15 Street, 61-866 Poznań, Poland

**Keywords:** breast cancer conservative surgery, surgical wound fluids, intraoperative radiation therapy, radiation induced bystander effect

## Abstract

In patients with breast cancer who undergo breast-conserving surgery (BCS), more than 90% of local recurrences occur in the same quadrant as the primary cancer. Surgical wound fluids (SWF) are believed to play a role in this process by inducing an inflammatory process in the scar tissue area. Despite strong clinical data demonstrating the benefits of intraoperative radiotherapy (IORT), the biological basis underlying this process remains poorly understood. Ionizing radiation (IR) directly affects cells by damaging DNA, thereby altering the cell phenotype. IR directly affects cancer cells and also influences unirradiated cells located nearby, a phenomenon known as the radiation-induced bystander effect (RIBE), significantly modifying the tumor microenvironment. We hypothesized that SWF obtained from patients after BCS and IORT would induce a radiobiological response (due to RIBE) in unirradiated cells, thereby modifying their phenotype. To confirm this hypothesis, breast cancer cells were incubated with SWF collected from patients after BCS: (1) without IORT (wound fluid (WF) group), (2) with IORT (radiotherapy wound fluid (RT-WF) group), and (3) WF with conditioned medium from irradiated cells (WF+RIBE group) and then subjected to microarray analysis. We performed gene set enrichment analysis to determine the biological processes present in these cells. This analysis showed that the RT-WF and WF+RIBE groups shared common biological processes, including the enhancement of processes involved in cell-cycle regulation, DNA repair, and oxidative phosphorylation. The WF group was characterized by overrepresentation of pathways involved in the INF-α and INF-γ response, inflammatory response, and the IL6 JAK/STAT3 signaling pathway. These findings show that MDA-MB-468 cells stimulated with surgical wound fluids obtained from patients who underwent BCS plus IORT and from cells stimulated with SWF plus RIBE share common biological processes. This confirms the role of the radiation-induced bystander effect in altering the biological properties of wound fluids.

## 1. Introduction

In patients with breast cancer treated with breast-conserving surgery (BCS), metastases and recurrences are the leading cause of cancer-related mortality [1]. Postoperatively, most patients undergo external beam radiotherapy (EBRT) to eradicate the remaining cancer cells after BCS, which tend to localize around the original tumor site [2]. Intraoperative radiation therapy (IORT), which involves localized dose escalation to eliminate these residual cancer cells while minimizing toxicity to the surrounding healthy tissue, can be used as an alternative to EBRT or as an adjuvant therapy in localized breast cancer. Studies have shown that the survival and toxicity outcomes achieved with IORT are comparable to EBRT [3,4,5]. Moreover, some reports have suggested that IORT may alter the microenvironment of the tumor bed, making recurrence less likely [6].

Surgical excision of the primary tumor induces a wound healing response and inflammatory processes that alter the local environment and stimulate the growth kinetics of the remaining cancer foci, thereby potentially promoting metastasis [7,8,9]. Studies using global transcriptome analysis of a panel of triple-negative breast cancer (TNBC) cell lines have found that stimulation with surgical wound fluid (SWF) significantly increases the expression of genes related to wound response, cytokine activity, and locomotory characteristics [10]. Belletti et al. [6] showed that IORT after BCS alters this effect, making the microenvironment less favorable for tumor cells. Those authors found that SWF from patients after BCS stimulates the proliferation, invasion, and migration of breast cancer cells, but administration of IORT abrogates this effect. Previously, we have conducted the quantitative analysis of SWF composition in breast cancer (BC) patients treated with BCS only, and BCS followed by IORT, and found significant changes based not only on the applied treatment but also on the molecular subtype of BC [11]. Moreover, our group showed that the radiation-induced bystander effect (RIBE) alters the biological activity of SWF after IORT [12,13]. However, the biological basis underlying this process is still not well-understood.

In this context, the aim of the present study was to assess the differential global transcriptome changes in a TNBC cell line (MDA-MB-468) after stimulation with SWF obtained from a series of patients with breast cancer treated with BCS or BCS plus IORT. In addition, we sought to verify whether RIBE plays a role in the altered biological activity of these wound fluids.

## 2. Results

### 2.1. Breast Cancer Cells Treated with Surgical Wound Fluids Are Associated with an Enhancement of Processes Associated with Tumorigenicity and Metastatic Potential

**Figure 1:**
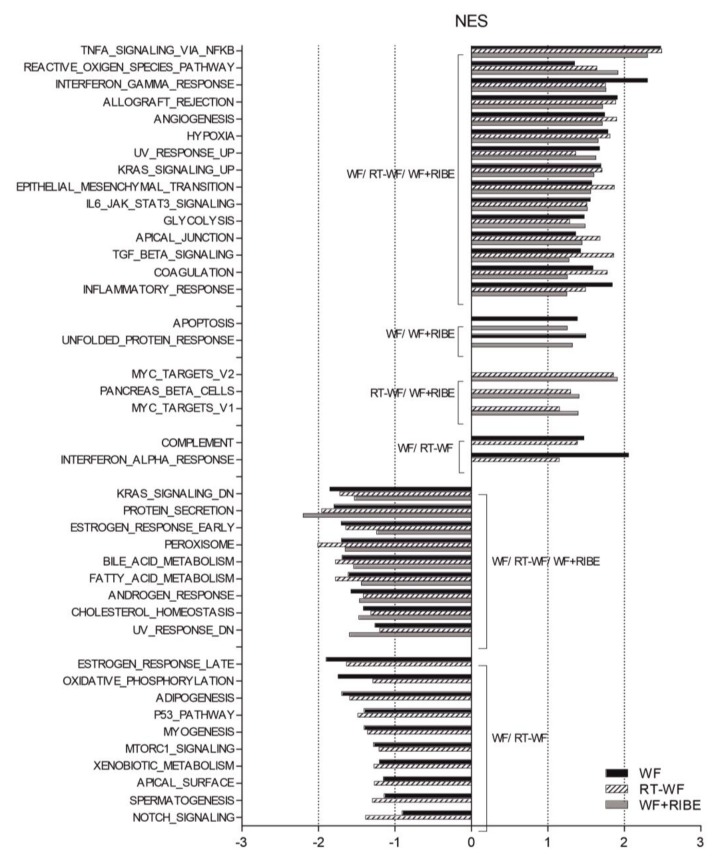
Breast cancer cells treated with surgical wound fluids are enriched in processes associated with tumorigenicity and metastatic potential. MDA-MB-468 cell lines were incubated with wound fluid (WF), radiotherapy wound fluid (RT-WF), and wound fluid with radiation-induced bystander effect (WF+RIBE) fluids for two days and subjected to microarray analysis. Each fluid was compared to controls to identify differentially expressed genes. Gene set enrichment analysis (GSEA) was then performed, revealing the biological processes engaged in cell response to stimulation with these different fluids. While changes in some biological processes were present in all three groups, some of these processes were present only in two groups (RT-WF and WF+RIBE).

To compare the transcriptome profiles of the MDA-MB-468 cells stimulated with the three different SWF mediums (WF, RT-WF, and WF+RIBE), we analyzed whole-genome expression using Affymetrix Human Gene 2.1 ST Array Strips. First, following the principles of Principal Component Analysis (PCA), the control cells (unstimulated) and treated cells (SWF) were segregated. The following selection criteria were assumed to indicate a significantly changed gene expression: expression fold change (FC) difference ≥ 2 with adjusted *p*-value ≤ 0.05. While the wound fluid (WF) group was highly segregated from the radiotherapy wound fluid (RT-WF) and WF-RIBE groups, the RT-WF and WF-RIBE cells were very similar to each other (Supplementary Figure S1). Based on the aforementioned selection, the highest gene expression for the RT-WF cells vs. controls was as follows: xanthine dehydrogenase (XDH); interleukin 22 receptor; alpha 2 (IL22RA2); cadherin 3; type 1 (CDH3); suppressor of cytokine signaling 3 (SOCS3); gamma-aminobutyric acid B receptor 2 (GABBR2); guanylate binding protein 2 interferon-inducible (GBP2); solute carrier family 12 (sodium/potassium/chloride transporter); membrane 2 (SLC12A2); colorectal cancer associated 2 (COLCA2); cardiomyopathy associated 5 (CMYA5); cysteine-rich secretory protein 3 (CRISP3), and KIAA1324 (Supplementary Figure S2). Some of these genes were also observed in the WF group vs. controls (CRISP3 COLCA2, SLC12A2, KIAA1324) and the WF+RIBE cells vs. controls (CMYA5, SLC12A2, COLCA2, CRISP3, KIA1324) (Supplementary Tables S3 and S4). RT-qPCR analysis was performed to validate the highly differentially expressed genes (Supplementary Table S5). Differentially expressed genes from the following comparisons: RT-WF vs. controls, WF vs. controls, WF+RIBE vs. controls, RT-WF vs. WF, and WF+RIBE vs. WF were assigned to gene set enrichment analysis (GSEA). This analysis revealed the involvement of a biological process engaged in cell response to stimulation with the different SWFs. Some biological processes were present in all three study groups, while others were observed in only two groups (Figure 1, Supplementary Tables S1–S3). The highly overrepresented biological processes present in all three groups were as follows: TNFA signaling via NFKB; reactive oxygen species pathway; interferon gamma response; allograft rejection; angiogenesis; hypoxia; epithelial to mesenchymal transition; IL6_JAK_STAT3 signaling; glycolysis; and inflammatory response. By contrast, the most underrepresented biological processes characteristic for these three groups were: KRAS signaling; protein secretion; estrogen response; peroxisome; fatty acid metabolism.

### 2.2. The Biological Processes in Cancer Cells Enriched in RT-WF and WF+RIBE-Treated Cells Are Similar and Related to Cell Proliferation, Cell Division, and DNA Damage Responses

**Figure 2:**
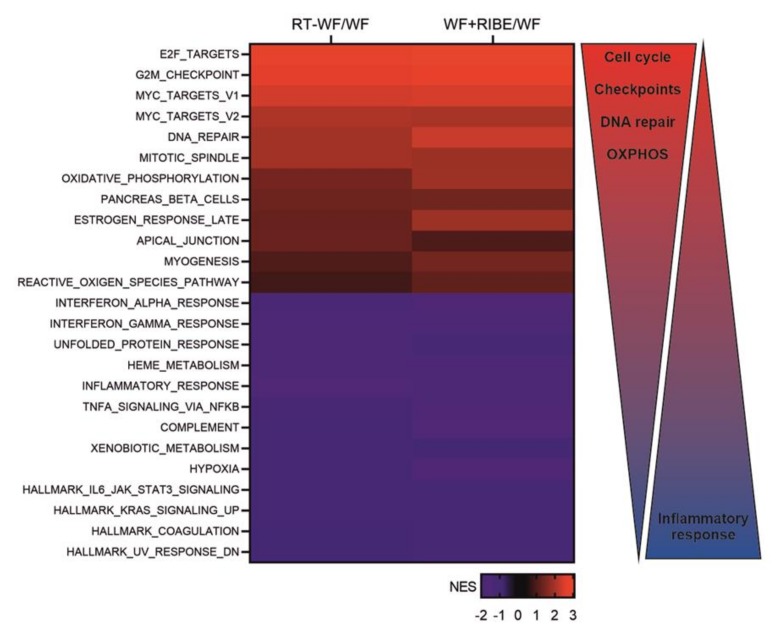
Biological processes enriched in RT-WF and WF+RIBE-treated cells are similar. MDA-MB-468 cell lines were incubated with WF, RT-WF, and WF+RIBE fluids for two days and subjected to microarray analysis. The fluids were compared to controls to identify differentially expressed genes. These genes were then subjected to gene set enrichment analysis (GSEA), which showed that the biological processes enriched in RT-WF and WF+RIBE-treated cells were similar and related to cell proliferation, division DNA damage response, and oxidative phosphorylation.

To elucidate the biological processes activated by IORT and to determine whether these processes are dependent on the RIBE, a GSEA analysis was conducted to compare the RT-WF-treated cells to the WF group and to compare the WF+RIBE-treated cells to the WF group. The biological processes that were over- and underrepresented in each group were then compared to each other. Processes with nominal *p* values ≤ 0.05 are shown in Supplementary Tables S4 and S5. Most of the processes that were significantly over-or underrepresented were the same for both groups. Based on normalized enrichment score (NES) values, a heat map showing common processes for RT-WF vs. WF and WF+RIBE vs. WF was generated (Figure 2).

**Figure 3:**
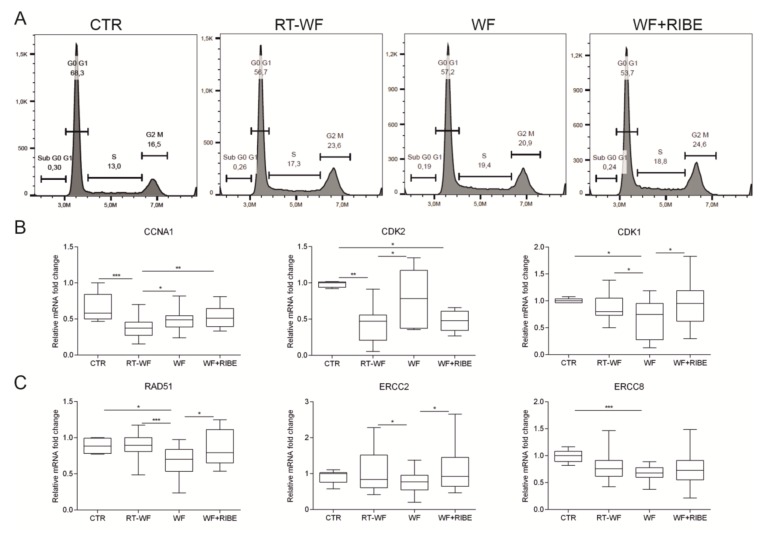
Surgical wound fluids affect the cell cycle and DNA damage response of the MDA-MB-468 cell line. MDA-MB-468 cell lines were incubated with WF, RT-WF, and WF+RIBE fluids for two days and subjected to cell-cycle (**A**) and gene-expression analysis (**B**,**C**). We confirmed the cell-cycle arrest at the G2M phase (**A**). RT-qPCR analysis revealed the expression of genes known to arrest the cycle in the S/G2 phase, thus confirming activation of the G2M checkpoint (**B**). We confirmed the upregulation of genes involved in nucleotide base repair mechanisms (NER): ERCC2, ERCC8; and homologous recombination (HR): RAD51 (**C**). The qPCR reaction was performed in the following samples: RT-WF (*n* = 22); WF (*n* = 21); WF+RIBE (*n* = 21); and controls (*n* = 8). The graphs represent relative mRNA fold changes ± standard deviation. * *p* < 0.05; ** *p* < 0.01; *** *p* < 0.001, **** *p* < 0.0001.

GSEA analysis suggested that most of the genes that were differentially expressed in these comparisons (RT-WF vs. WF and WF+RIBE vs. WF) were likely involved in cell proliferation, division, DNA damage response, and metabolism, as follows: E2F targets; G2M checkpoint; MYC targets; DNA repair; mitotic spindle; oxidative phosphorylation. Underrepresented processes responsible for an inflammatory response were: interferon alpha response; interferon gamma response; inflammatory response; TNF alpha signaling via NFκB; IL6 JAK/STAT3 signaling (Supplementary Table S4 and S5). These results indicate that the biological processes involved in cell proliferation and division are overrepresented in MDA-MB-468 cells treated with RT-WF or WF+RIBE compared to those treated with WF alone. GSEA enrichment plots were used to visualize those results (E2F targets, G2M checkpoint, mitotic spindle—Figure 2). Cell-cycle analysis was performed to confirm these findings. We confirmed the cell-cycle arrest at the G2M phase (Figure 3A). Using RT-qPCR, we also confirmed activation of the G2M checkpoint by analyzing the expression of genes known to arrest the cycle in the S/G2 phase (Figure 3B). The expression of cyclin A1 (CCNA1) was significantly decreased in RT-WF-treated cells compared to controls and to WF-treated cells. While the WF+RIBE-treated cells had a lower proliferation capacity than RT-WF-treated cells, and a higher percentage of G2M phase positive cells, cyclin A expression was significantly higher in the WF+RIBE-treated cells. Expression of cyclin-dependent kinase 2 (CKD2), which is highly expressed in the G1/S phase and downregulated as cells reach the G2/M checkpoint, was lower in WF-treated cells. This lower expression of CKD2 vs. control cells was observed only in RT-WF and WF+RIBE-treated cells. We also confirmed increased expression of cyclin-dependent kinase 1 (CDK1) in the RT-WF and WF+RIBE groups compared to WF-treated cells.

The G2M checkpoint, which prevents cells from initiating mitosis, is activated when the cell DNA is damaged as cells proceed to the G2 phase without completing DNA repair in the S or G1 phase. The GSEA revealed upregulation of DNA repair in the RT-WF group vs. WF (NES = 1.949, Supplementary Table S4) and in the WF+RIBE vs. WF (NES = 2.319, Supplementary Table S4). Detailed analysis of gene expression within this process revealed upregulation of genes involved in nucleotide excision repair mechanisms (NER): ERCC2, ERCC8; and upregulation of homologous recombination (HR): RAD51. To verify those results, RT-qPCR was performed on a larger group of samples (*n* = 20), showing that expression of ERCC2 and RAD51 was upregulated in RT-WF and WF+RIBE-treated cells compared to the WF group. ERCC8 expression was also higher (but not significantly) in the RT-WF and WF+RIBE samples compared to the WF-treated cells. (Figure 3C).

### 2.3. Surgical Wound Fluids Alter the Metabolism of the MDA-MB-468 Cell Line

**Figure 4:**
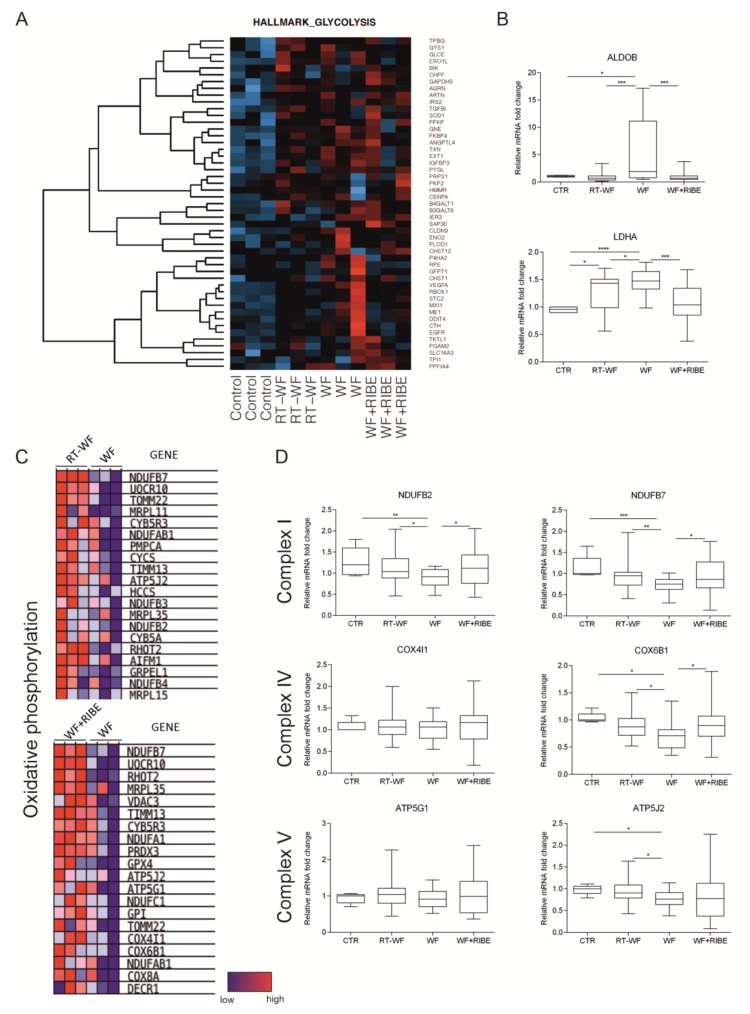
Surgical wound fluids affect the metabolism of the MDA-MB-468 cell line. Microarray analysis revealed that MDA-MB-468 cells treated with RT-WF, WF, or WF+RIBE fluids are significantly enriched in glycolytic metabolism compared to untreated cells (controls) (**A**). This enriched glycolysis was confirmed by RT-qPCR analysis (**B**). However, microarray data from comparisons (RT-WF vs. WF and WF+RIBE vs. WF) indicate that MDA-MB-468 cells treated with RT-WF or WF+RIBE are characterized by enhanced oxidative phosphorylation (**C**). Those results were confirmed by RT-qPCR analysis (**D**). The qPCR reaction was performed the following samples: RT-WF (*n* = 22), WF (*n* = 21), WF+RIBE (*n* = 21), and controls (*n* = 8). The graphs represent relative mRNA fold changes ± standard deviation. * *p* < 0.05; ** *p* < 0.01; *** *p* < 0.001, **** *p* < 0.0001.

One of the hallmarks of cancer is a metabolic switch from glycolysis and oxidative phosphorylation to aerobic glycolysis with high lactate production. In aerobic glycolysis, pyruvate is converted to lactic acid by lactic acid dehydrogenase-A (LDHA) [14]. The microarray analysis revealed that MDA-MB-468 cells treated with all three SWF mediums were significantly enriched in glycolytic metabolism compared to controls (untreated) (Table 1, Figure 4A). Fatty acid metabolism was significantly decreased in all cells treated with SWF. However, microarray data comparing the RT-WF and WF+RIBE-treated cells to the WF group showed that MDA-MB-468 cells treated with RT-WF or WF+RIBE were characterized by enhanced oxidative phosphorylation (Table 1, Figure 4D). To verify those findings, we performed an RT-qPCR assay on a larger sample (*n* = 20) to measure LDHA expression, finding that expression of LDHA was increased in all three treated cells (RT-WF, WF and WF+RIBE) versus controls. Moreover, expression of LDHA was significantly higher in WF-stimulated cells than in cells treated with RT-WF and WF+RIBE (Figure 4B).

While GSEA analysis comparing the treated cells (RT-WF/WF/WF+RIBE) to controls revealed that cells stimulated with SWF are enriched with glycolysis, the same analysis showed that RT-WF and WF+RIBE-treated cells were enriched with oxidative phosphorylation when compared to WF-treated cells (Figure 4C). To verify those findings, we performed mRNA analysis on selected genes based on microarray analysis in an expanded sample (*n* = 20 for each group). The expression of genes belonging to complex I (NDUFB2, NDUFB7), complex IV (COX4I1, COX6B1), and complex V (ATP5G1, ATP5J2) of the mitochondrial electron transport chain was analyzed by RT-qPCR (Figure 4D). This analysis showed that expression of NDUFB2, NDUFB7, COX6B1, and ATP5J2 was lower in SWF-treated cells than in controls. In addition, the expression of NDUFB2, NDUFB7, COX6B1 was significantly lower in WF-treated cells than in cells treated with RT-WF and WF+RIBE, while ATPJ2 expression was significantly lower in WF-treated cells than in RT-WF-treated cells. We did not find any differences in COX4I1 or ATP5G1 expression among the groups.

## 3. Discussion

The main aim of this study was to assess the global transcriptome changes in a TNBC cell line after incubation with surgical wound fluids obtained from breast cancer patients after BCS and after BCS plus IORT. In addition, we performed microarray analysis to determine whether SWF obtained from patients who underwent IORT act as a mediator (due to the bystander effect), thereby altering the biology of unirradiated cells. In a previous study, our group investigated the link between RIBE and the anti-cancer properties of WF in the surgical cavity after IORT, demonstrating that IORT induces the release of bystander factors that mediate the radiobiological response in breast cancer cells [12]. Moreover, in that same study, we confirmed that the bystander effect induced by IORT abrogates the role of SWF in stimulating cancer stem cell phenotype and epithelial-to-mesenchymal transition (EMT) in breast cancer cells [13]. The findings of the present study provide additional insight into the specific mechanisms regulating these processes. In the present study, we showed that (1) surgical wound fluids collected from breast cancer patients activate numerous biological pathways considered to be hallmarks of cancer (angiogenesis, hypoxia, EMT, activation of IL6/JAK/STAT3 pathway, inflammatory response) in the MDA-MB-468 cell line, (2) both IORT and RIBE alter the properties of surgical wound fluids, and both of these activate the same pathways in the MDA-MB-468 cell line, (3) the biological pathways activated by RT-WF and WF+RIBE mediums in MDA-MB-468 cells indicate that these cells have lower tumorigenic potential than cells stimulated with SWF obtained from patients treated with BCS alone.

The wound-healing process, together with surgically-induced inflammation, stimulate the growth of residual breast cancer cells after tumor excision [15]. Local recurrence after surgery is particularly common in tumors that overexpress HER2. Studies have also shown that SWF contains growth factors that induce proliferation of HER2-positive breast cancer [16]. However, as the TARGIT studies have shown, IORT significantly inhibits the stimulatory effect of SWF on tumor cells in vitro, which may occur by the direct effect of IR (cell killing) and by modulating the tumor microenvironment [17]. IR directly affects the cells by damaging DNA, thereby changing the cell phenotype. However, in addition to the direct effects of IR, its action may also be observed in unirradiated cells located in close proximity to the irradiated cells (i.e., the bystander effect). This effect can be observed in many cell types at different biological levels (DNA damage, genomic instability, malignant transformation, and cell death). The RIBE significantly modifies the tumor microenvironment and although this effect is most commonly observed at low and medium doses, it may also play a significant role in high dose irradiation [12]. It is known that irradiation changes the profile of secreted cytokines. We have previously shown that composition of SWF from BCS and IORT patients differs significantly based on cytokine and growth-factor concentrations [11].

In the current study, we used global transcriptome analysis to confirm that SWF collected from breast cancer patients is associated with increased tumorigenicity and metastatic potential of MDA-MB-468 cells. Compared to untreated cells, the cells incubated with all three fluid groups (RT-WF, WF, WF+RIBE) were highly enriched with biological processes associated with tumorigenicity and metastasis, including the following: angiogenesis; hypoxia; EMT; IL6/JAK/STAT pathway; glycolysis; and inflammatory response. The RT-WF and WF+RIBE-treated cells were highly correlated in terms of the biological processes that were enriched, indicating a lower tumorigenic and metastatic potential in these cells compared to cells stimulated with WF alone.

The GSEA analysis suggested that most of the genes that were differentially expressed in the comparisons (RT-WF vs. WF, and WF+RIBE vs. WF) were likely involved in cell proliferation, cell division, DNA damage response, and metabolism, as follows: E2F targets; G2M checkpoint; MYC targets; DNA repair; mitotic spindle; and oxidative phosphorylation. By contrast, the underrepresented processes were mainly those responsible for inflammatory response, as follows: interferon alpha response, interferon gamma response, inflammatory response, TNF alpha signaling via NFκB, IL6 JAK/STAT3 signaling. DNA is the primary target of cell damage. Damage to DNA prevents cell-cycle progression by activating DNA damage response (DDR) mechanisms, a complex system that determines the cellular outcome of DNA damage. DDR activates effector signaling pathways such as cell-cycle checkpoints (responsible for accurate DNA synthesis and cell division), DNA repair, and cell death. The E2F transcription factor family plays an important role in cell-cycle progression. Ren et al. identified a group of genes regulated by E2F that encode DNA damage checkpoint and repair pathways, chromatin condensation, chromosome segregation, and the mitotic spindle checkpoint [18]. DNA damage sensor proteins recognize specific DNA lesions and then initiate the DDR mechanism. One of the earliest events after DDR is activation of the ataxia-telangiectasia mutated (ATM) protein, which targets the histone protein, H2AX. This protein is phosphorylated at the double-strand break (DSB) sites and is a marker of DDR. In a previous study [12], we measured the level of DSBs, induction of apoptosis, and the changes in the expression of genes related to DNA damage repair. In that study, we found that stimulating cells with RT-WF and with WF+RIBE induced DSBs and increased the expression of DNA damage repair-related genes, which was not observed after stimulation with WF alone. Two DNA repair mechanisms—NER and HR—were highly activated after RT-WF and WF+RIBE stimulation [12]. In that study, a detailed analysis of gene expression within the biological DNA repair processes that were enriched in cells treated with RT-WF and WF+RIBE versus WF alone revealed upregulation of genes involved in NER (ERCC2, ERCC8) and HR (RAD51). Those finding suggest that IORT induces changes in the tumor microenvironment that mediate the genotoxic effect of IR [12].

The metabolic switch is one of the hallmarks of cancer [19]. The Warburg effect refers to the observation that cancer cells tend to prefer aerobic glycolysis as the main mode of glucose metabolism versus oxidative phosphorylation. However, since aerobic glycolysis produces only two ATP molecules from a glucose molecule, cancer cells need to increase their uptake of glucose from the microenvironment to meet their energy requirements. To maintain homeostasis in the microenvironment, those cancer cells secrete more lactic acid [14,20], which then decreases the pH in the tumor microenvironment, thus enhancing the metastatic potential of those cancer cells [21]. Chang et al. observed that high glycolysis rates limit access of tumor infiltrating lymphocytes (TIL) to the cancer cells, thereby suppressing the function of TIL to eliminate cancer cells [22]. Enhanced glycolysis and production of lactate (aerobic glycolysis) are considered hallmarks of cancer [19]. In this study we demonstrated that incubation of MDA-MB-468 cells with SWF increases glycolytic metabolism in these cells. We have also confirmed the increased expression of LDHA (Figure 4B). Thus, we have confirmed that SWF are capable of triggering a switch in cell metabolism to aerobic glycolysis. These results are consistent with reports showing that SWF, which are rich in inflammatory factors, could stimulate residual cancer cell growth and increase their metastatic potential [15,16]. Although all SWF stimulate aerobic glycolysis in MDA-MB-468 cells, our results show that LDHA expression was significantly lower in RT-WF and WF+RIBE-stimulated cells. Moreover, microarray analysis revealed a greater enrichment of oxidative phosphorylation in RT-WF and WF+RIBE-treated cells compared to cells treated with WF alone. Those results were also confirmed by RT-qPCR analysis showing that genes belonging to complex I, IV, and V of the mitochondrial electron transport chain were more highly expressed in RT-WF and WF+RIBE-treated cells than in WF-treated cells. These findings indicate that the RT-WF and WF+RIBE mediums have less tumorigenic potential then WF alone. Lu et al. found that mitochondrial oxidative metabolism is a suppressor of metastasis and that the Warburg effect not only gives cancer cells the energy needed to grow, but also facilitates metastatic progression [23]. One of the drivers associated with cancer progression is EMT, during which cancer cells increase their metastatic potential, their cancer stem cell phenotype, and their therapeutic resistance [24]. The transcription factor SNAIL is a central driver of EMT, with studies showing that pro-metastatic SNAIL attenuates oxidative phosphorylation [25]. SNAIL binds to the promoters of cytochrome c oxidase (COX) subunits and inhibits their expression. Increased SNAIL expression decreases COX expression, thereby repressing mitochondrial respiration. Therefore, it can be postulated that mitochondrial oxidative metabolism is downregulated while cells undergo metastasis.

We have previously demonstrated that stimulation with WF increases markers of EMT (CDH2, SNAIL, and VIM) in MDA-MB-468 cells more than stimulation with RT-WF or WF+RIBE (12). In the present study, we have shown that glycolytic marker expression was increased in SWF-treated cells while the expression of genes belonging to complex I (NDUFB2, NDUFB7), complex IV (COX6B1), and complex V (ATP5J2) of the mitochondrial electron transport chain was significantly lower in WF-treated versus RT-WF or WF+RIBE-treated cells. Research has shown that the Warburg effect is implicated in resistance to cytotoxic stress, including IR and chemotherapy [26,27]. Therefore, any treatment that decreases glycolysis after radiotherapy may increase cell sensitivity to additional treatment with radiotherapy and chemotherapy.

## 4. Materials and Methods

### 4.1. Wound Fluid Collection

Wound fluids were collected from patients who underwent BCS for breast cancer at the Greater Poland Cancer Centre (Poznan, Poland). Patient informed consent was obtained following previously described procedures [12,13]. The study was approved by the Bioethics Committee of the Poznan University of Medical Sciences (study number: 756/16 from 16 June 2016). All the experiments were conducted in accordance with Bioethics Committee guidelines. Wound fluids were collected from patients treated with BCS plus IORT (*n* = 22; RT-WF group) and from a second group of patients treated with BCS alone without IORT (*n* = 21; WF group). The mean age of these patients was 58.3 ± 11.3 years (RT-WF group) and 59.6 ± 10.6 years (WF group). Moreover, all patients were chosen based on histological subtype (all were classified as NST) and similar TNM status: T1, N0, or N1. The SWF in groups correspond to liminal A, luminal B, HER2 positive and TNBC molecular subtype of BC patients.

### 4.2. Cell Culture

All experiments were conducted on the MDA-MB-468 breast cancer cell line (estrogen receptor (ER)-negative, progesterone receptor (PR)-negative, and HER2 negative)( ATCC, Rockville, MD). Cells were cultured in a humidified atmosphere with 5% carbon dioxide at 37 °C (BINDER, Tuttlingen, Germany) in Dulbecco modified Eagle medium (Biowest, Nuaillé, France) supplemented with 10% fetal bovine serum (Biowest, France) and 1% penicillin/streptomycin 10,000 U/mL (Merck Millipore, Darmstadt, Germany).

### 4.3. Radiation-Induced Bystander Effect Medium (RIBE)

RIBE medium was collected as described previously [12,13]. Shortly, MDA-MB-468 cells were irradiated in suspension. A total dose of 10 Gy (which corresponds to 10 Gy in was administered at approximately 2.5 Gy/min using GammaCell^®^ 1000 Elite (BestTheratronics Ltd., Ottawa, Canada) using a Caesium-137 source. After irradiation, the cells were cultured for 24 h and then the RIBE medium was collected, sterile-filtered, and stored at −80 °C. In order to analyze the effect of RIBE without any perturbing factor, we decided to perform the analysis on the medium collected from the corresponding cells.

### 4.4. Cell Treatment

MDA-MB-468 cells were incubated with SWF obtained from the two study groups (RT-WF and WF groups). In all cases, the SWF was conditioned in DMEM without fetal bovine serum (FBS). The WF and RT-WF groups both contained 10% WF, which were conditioned in DMEM without FBS, as stated above. A third group of cells was conditioned in 10% RIBE and 10% WF (WF+RIBE).

### 4.5. RNA Isolation

Total RNA was isolated 48 h after stimulation with the study fluids using Direct-zol™ RNA MiniPrep (Zymo Research; Irvine, CA, USA) according to the manufacturer’s instructions. RNA was eluted in 25 µL of DEPC-treated H2O (Sigma, Aldrich, Merck KGaA; Darmstadt, Germany). The total RNA concentration was determined spectrophotometrically by measuring absorbance at 260 nm (NanoDrop, Thermo Scientific; Waltham, MA, USA). For microarray analysis, the quality and integrity of RNA was checked in a Bioanalyzer 2100 (Agilent Technologies, Inc., Santa Clara, CA, USA). For the microarray experiments, 100 ng of total RNA was used. One µg of total RNA was subjected to reverse transcription using the iSCRIPT kit (BioRad, Hercules, CA, USA) according to the manufacturer’s instructions.

### 4.6. Microarray Expression Study

The microarray study was carried out as described in previous studies [28,29,30]. For microarray analysis, three samples from different SWF were chosen for the BCS and IORT group. Briefly, total RNA (100 ng) from each sample was subjected to two rounds of sense cDNA amplification, biotin labeling, and cDNA fragmentation using the GeneChip Whole Transcript (WT) PLUS Reagent Kit (Affymetrix Inc., Santa Clara, CA, USA). Biotin-labeled fragments of cDNA (5.5 μg) were hybridized by the Affymetrix Human Gene 2.1 ST ArrayStrip (20 h, 48 °C). After hybridization, the microarrays were washed and stained using the Affymetrix GeneAtlas Fluidics Station (Affymetrix, Santa Clara, CA, USA). The array strips were scanned on the Imaging Station from GeneAtlas System (ThermoFisher Scientific, Waltham, MA, USA). Preliminary analysis of the scanned chips was performed using the Affymetrix GeneAtlas Operating Software (Affymetrix, Santa Clara, CA, USA), where the quality of detected signals was verified. The CEL files were imported for downstream data analysis.

### 4.7. Microarray Data Analysis

All analyses were performed using BioConductor software with the relevant Bioconductor libraries, based on the statistical R programming language (www.bioconductor.org). The Robust Multiarray Average (RMA) normalization algorithm implemented in the “Affy” library was used for normalization, background correction, and calculation of the expression values of all examined genes [31]. Biological annotation was taken from the BioConductor “oligo” package, where the annotated data frame object was merged with the normalized data set, leading to a complete gene data table [32]. Differential expression and statistical assessment were determined by applying the linear models for microarray data implemented in the “limma” library [33]. The selection criteria for significantly changed gene expression were based on fold differences higher than absolute 2 and adjusted *p*-value < 0.05. The result of this selection was presented as a volcano plot, showing the total number of up- and down-regulated genes. Raw data files were also deposited in the gene expression omnibus (GEO) repository at the National Center for Biotechnology Information (http://www.ncbi.nlm.nih.gov/geo/) under the GEO accession number GSE135204. The whole sets of differentially expressed genes from all comparisons were subjected to principal component analysis (PCA), as well as correlation coefficient analysis. For this purpose, “pca3d” and “corrplot” Bioconductor libraries were used [34,35].

### 4.8. Gene Set Enrichment Analysis (GSEA)

GSEA was used to determine enrichment or depletion in gene expression between two biological groups within a priori defined gene sets (GO terms, pathways). The method uses the Kolmogorov–Smirnov (K-S) statistical test to identify significantly enriched or depleted groups of genes [36]. Normalized fold change values from all of the genes presented on the microarray were log2 transformed and ordered. Then, a predefined gene set from the Hallmark database (from the Molecular Signatures Database) was selected [37]. Genes belonging to the selected set were ranked according to the difference in their expression level using a signal-to-noise ratio with 1000 permutations. By walking down the ranked list of genes, the enrichment score (ES) was calculated for each selected gene set [38] and these scores were normalized by their gene set size, with false positives corrected by the false-discovery rate technique.

### 4.9. Real-Time Quantitative PCR RT-qPCR

To validate the microarray analysis, the expression of selected markers was analyzed using the synthesized PRIMEPCR plate (BioRad, CA) with the Fast Start Essential DNA Green Master (Roche, Basel, Switzerland) on LightCycler96 (Roche, Basel, Switzerland) according to the manufacturer’s instructions. Expression of LDHA and genes belonging to mitochondrial electron transport chain was evaluated using specific primers and UPL probes designed with the RealTime Ready Assay Design and Roche Probes Master kit (Roche, Basel, Switzerland) on LightCycler96 (Roche, Basel, Switzerland) according to the manufacturer’s instructions. The qPCR reaction was performed on 22 RT-WF samples, 21 WF samples, 21 WF+RIBE samples, and eight unstimulated samples as controls.

### 4.10. Flow Cytometry

Cell-cycle analysis (48h after adding SWF) was performed with propidium iodide (P1304MP, Thermo Fisher Scientific, MA, USA). Cells were analyzed with a flow cytometer (Cytoflex, Beckman Coulter, CA, USA). Data were analyzed using FlowJo software (FlowJo v10; LLC, Ashland, OR, USA).

### 4.11. Statistical Analysis

Statistical analyses were performed using the GraphPad Prism software program, v.8 (GraphPad Software, Inc., La Jolla, CA, USA). Data were examined using one-way ANOVA analysis with Tukey’s post-hoc test. *p* < 0.05 was considered to indicate a statistically significant difference.

## 5. Conclusions

Global transcriptome profiling revealed common biological processes in cells treated with RT-WF and WF+RIBE. This study shows that processes involved in cell-cycle checkpoint and DNA repair processes are higher in RT-WF and WF+RIBE-treated cells than in cells stimulated with WF alone. This study also reveals a metabolic shift from glycolytic to oxidative phosphorylation in RT-WF and WF+RIBE-treated cells versus cells treated with WF alone. These findings demonstrate that changes in the tumor microenvironment induced by irradiation increase the anti-tumor effects of treatment.

## Figures and Tables

**Table 1:** Metabolic pathways revealed by GSEA analysis in MDA-MB-468 cells.

Pathway	Comparisons	NES	NOM *p*-Val	FDR *q*-Val
Glycolysis	RT-WF vs. CTR	1.29	0.035	0.074
	WF vs. CTR	1.48	0.002	0.017
	WF+RIBE vs. CTR	1.49	0.002	0.024
Fatty acid metabolism	RT-WF vs. CTR	−1.78	0.000	0.004
	WF vs. CTR	−1.61	0.000	0.004
	WF+RIBE vs. CTR	−1.44	0.009	0.042
Oxidative phosphorylation	RT-WF vs. WF	1.42	0.005	0.043
	WF+RIBE vs. WF	1.88	0.000	0.000

## Supplementary Materials

The following are available online at https://www.mdpi.com/1422-0067/21/3/1159/s1.

## Abbreviations

ATP5G1	ATP synthase membrane subunit c locus 1
ATP5J2	ATP synthase membrane subunit f
BC	breast cancer
BCS	breast conserving surgery
COX4I1	cytochrome c oxidase subunit 4I1
COX6B1	cytochrome c oxidase subunit 6B1
EBRT	external beam radiotherapy
EMT	epithelial to mesenchymal transition
ER	estrogen receptor
ES	enrichment score
FBS	fetal bovine serum
FC	fold change
GEO	Gene Expression Omnibus
GSEA	gene set enrichment analysis
HR	homologous recombination
IORT	intraoperative radiation therapy
IR	ionizing radiation
KEGG	Kyoto Encyclopedia of Genes and Genomes
LDH	lactic acid dehydrogenase
NDUFB2	NADH:ubiquinone oxidoreductase subunit B2
NDUFB7	NADH:ubiquinone oxidoreductase subunit B7
NER	nucleotide excision repair
NES	normalized enrichment score
PR	progesterone receptor
RIBE	radiation induced bystander effect
RT-WF	surgical wound fluids collected from patients after breast conserving surgery followed by IORT
SWF	surgical wound fluids
TNBC	triple-negative breast cancer
WF	surgical wound fluids collected from patients after breast conserving surgery
