# Supplementary material for: Surgical Wound Fluids from Patients with Breast Cancer Reveal Similarities in the Biological Response Induced by Intraoperative Radiation Therapy and the Radiation-Induced Bystander Effect—Transcriptomic Approach
Source: Int J Mol Sci. 2020 Feb 10;21(3):1159. doi: 10.3390/ijms21031159 (PMC7037222; doi:10.3390/ijms21031159)
Supplement: Supplementary file 1 [file ijms-21-01159-s001.pdf]

Supplementary Materials:

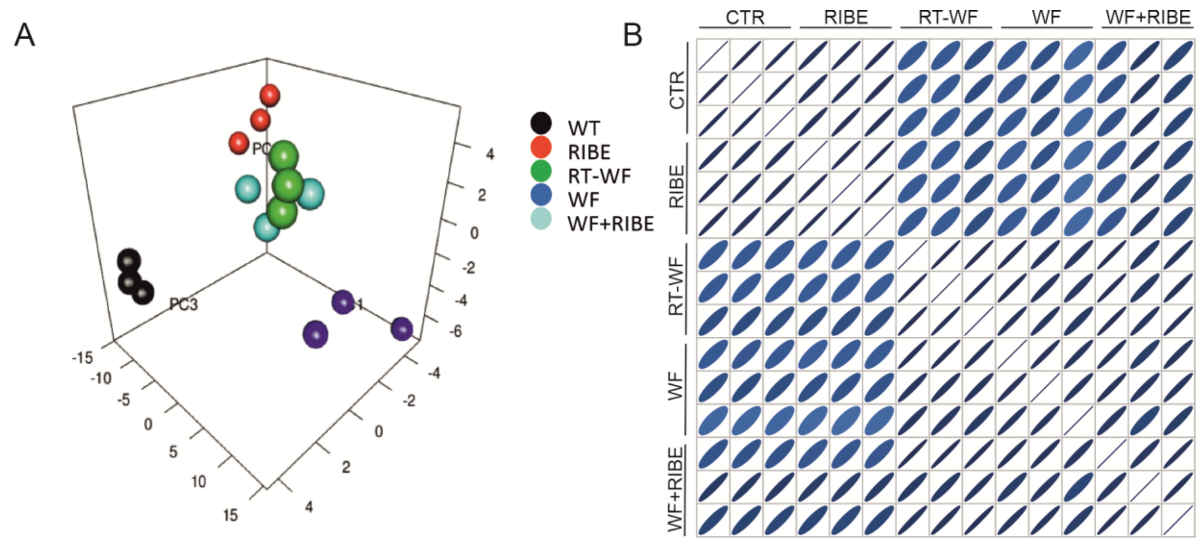

Figure S1. *Principal Component Analysis (PCA) and corplot.* PCA and corplot analysis indicated a segregation between control cells (unstimulated) and cells treated with the study surgical wound fluids. We assumed the following selection criteria for significantly changed gene expression: an expression fold change (FC) difference ≥ 2 with adjusted p value ≤ 0.05. While the WF group was highly segregated from the RT-WF and WF-RIBE groups, the RT-WF and WF-RIBE groups were very similar to each other.

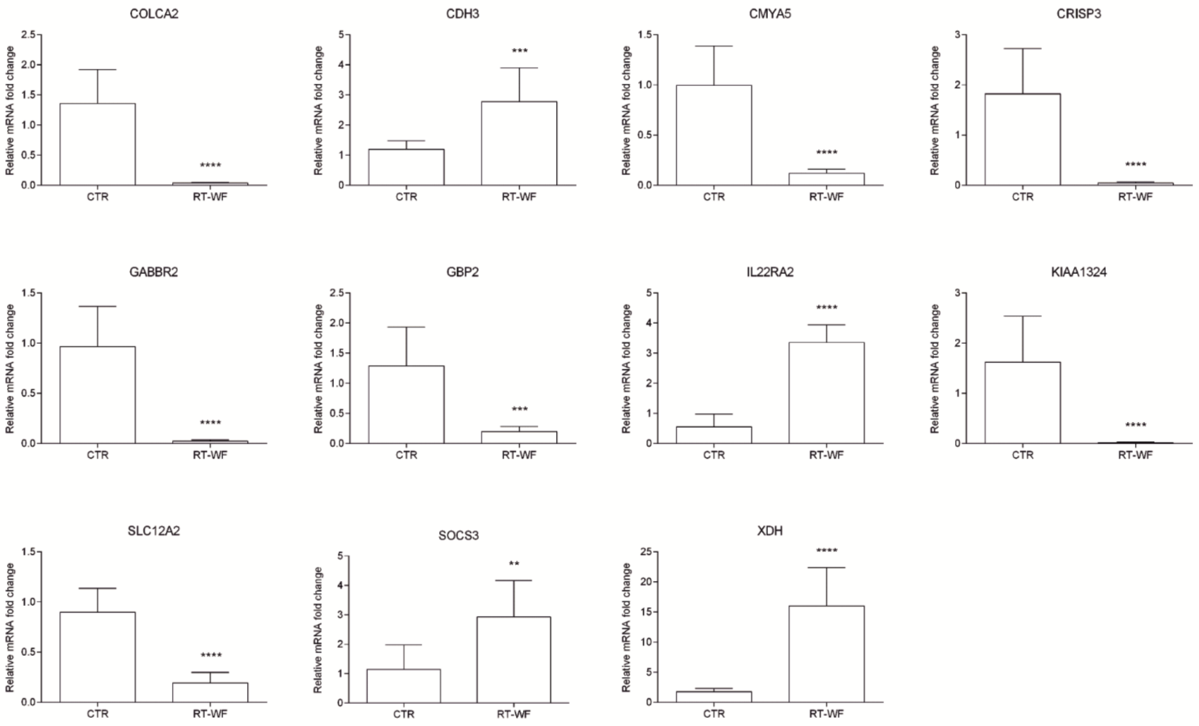

	Microarray CTR/RT-WF		RT-qPCR CTR/RT-WF	
Name	Fold change	P value	Fold change	p-value
COLCA2	4,67	p<0,001	35,03	p<0,0001
CDH3	-2,70	p<0,05	-2,32	p<0,001
CMYA5	4,74	p<0,01	9,63	p<0,0001
CRISP3	4,94	p<0,0001	40,51	p<0,0001
GABBR2	3,02	p<0,001	42,39	p<0,0001
GBP2	3,53	p<0,05	6,51	p<0,001
IL22RA2	-3,35	p<0,05	-6,01	p<0,0001
KIAA1324	7,73	p<0,001	106,96	p<0,0001
SLC12A2	3,99	p<0,001	4,63	p<0,001
SOCS3	-2,58	p<0,01	-6,10	p<0,01
XDH	-5,33	p<0,05	-8,99	p<0,0001

Figure S2. *Validation of microarray analysis by RT-qPCR.* The qPCR reaction was performed on 22 RT-WF samples and 8 CTR samples The Graphs represents relative mRNA fold changes ± standard deviation. * $p < 0.05$; ** $p < 0.01$; *** $p < 0.001$, **** $p < 0.0001$. Table shows fold change and p-value for both microarray and RT-qPCR analysis for CTR vs. RT-WF comparison.

Table S1. GSEA analysis of RT-WF stimulated vs CTR MDA-MB-468 cell line.

Pathway	NES	NOM *p*-val	FDR *q*-val	Enriched in Population
TNFA_SIGNALING_VIA_NFKB	2,489	0,000	0,000	RT-WF
ALLOGRAFT_REJECTION	1,886	0,000	0,000	RT-WF
ANGIOGENESIS	1,898	0,000	0,001	RT-WF
MYC_TARGETS_V2	1,857	0,000	0,001	RT-WF
COAGULATION	1,775	0,000	0,001	RT-WF
HYPOXIA	1,814	0,000	0,001	RT-WF
TGF_BETA_SIGNALING	1,860	0,000	0,001	RT-WF
EPITHELIAL_MESENCHYMAL_TRANSITION	1,869	0,000	0,001	RT-WF
INTERFERON_GAMMA_RESPONSE	1,753	0,000	0,001	RT-WF
KRAS_SIGNALING_UP	1,709	0,000	0,002	RT-WF
APICAL_JUNCTION	1,682	0,000	0,003	RT-WF
REACTIVE_OXIGEN_SPECIES_PATHWAY	1,639	0,009	0,004	RT-WF
IL6_JAK_STAT3_SIGNALING	1,509	0,002	0,012	RT-WF
INFLAMMATORY_RESPONSE	1,491	0,000	0,014	RT-WF
COMPLEMENT	1,384	0,011	0,038	RT-WF
UV_RESPONSE_UP	1,363	0,014	0,042	RT-WF
PANCREAS_BETA_CELLS	1,299	0,126	0,070	RT-WF
GLYCOLYSIS	1,286	0,035	0,074	RT-WF
INTERFERON_ALPHA_RESPONSE	1,147	0,174	0,192	RT-WF
MYC_TARGETS_V1	1,152	0,135	0,194	RT-WF
PROTEIN_SECRETION	−1,956	0,000	0,001	CTR
PEROXISOME	−2,009	0,000	0,002	CTR
BILE_ACID_METABOLISM	−1,829	0,000	0,003	CTR
FATTY_ACID_METABOLISM	−1,778	0,000	0,004	CTR
KRAS_SIGNALING_DN	−1,719	0,002	0,006	CTR
ESTROGEN_RESPONSE_LATE	−1,634	0,000	0,010	CTR
ESTROGEN_RESPONSE_EARLY	−1,641	0,000	0,011	CTR
ADIPOGENESIS	−1,590	0,000	0,014	CTR
P53_PATHWAY	−1,483	0,006	0,038	CTR
HEME_METABOLISM	−1,461	0,008	0,043	CTR
ANDROGEN_RESPONSE	−1,413	0,034	0,060	CTR
NOTCH_SIGNALING	−1,381	0,093	0,072	CTR
MYOGENESIS	−1,359	0,018	0,083	CTR
CHOLESTEROL_HOMEOSTASIS	−1,314	0,083	0,111	CTR
OXIDATIVE_PHOSPHORYLATION	−1,287	0,065	0,120	CTR
SPERMATOGENESIS	−1,294	0,061	0,122	CTR
APICAL_SURFACE	−1,270	0,122	0,123	CTR
XENOBIOTIC_METABOLISM	−1,273	0,046	0,126	CTR
UV_RESPONSE_DN	−1,198	0,129	0,183	CTR
APOPTOSIS	−1,202	0,130	0,186	CTR
MTORC1_SIGNALING	−1,208	0,126	0,188	CTR

Table S2. GSEA analysis of WF stimulated vs CTR MDA-MB-468 cell line.

**Pathway**	**NES**	**NOM *p*-val**	**FDR *q*-val**	**Enriched in Population**
TNFA_SIGNALING_VIA_NFKB	2,477	0,000	0,000	WF
INTERFERON_GAMMA_RESPONSE	2,303	0,000	0,000	WF
INTERFERON_ALPHA_RESPONSE	2,055	0,000	0,000	WF
INFLAMMATORY_RESPONSE	1,841	0,000	0,001	WF
ALLOGRAFT_REJECTION	1,909	0,000	0,001	WF
HYPOXIA	1,784	0,000	0,001	WF
ANGIOGENESIS	1,743	0,000	0,002	WF
KRAS_SIGNALING_UP	1,692	0,000	0,003	WF
UV_RESPONSE_UP	1,674	0,002	0,004	WF
COAGULATION	1,589	0,000	0,006	WF
EPITHELIAL_MESENCHYMAL_TRANSITION	1,573	0,000	0,008	WF
IL6_JAK_STAT3_SIGNALING	1,553	0,004	0,009	WF
UNFOLDED_PROTEIN_RESPONSE	1,497	0,002	0,015	WF
COMPLEMENT	1,472	0,009	0,016	WF
GLYCOLYSIS	1,475	0,002	0,017	WF
TGF_BETA_SIGNALING	1,426	0,044	0,024	WF
APOPTOSIS	1,385	0,013	0,032	WF
APICAL_JUNCTION	1,362	0,008	0,039	WF
REACTIVE_OXIGEN_SPECIES_PATHWAY	1,350	0,079	0,041	WF
HEME_METABOLISM	1,242	0,077	0,101	WF
IL2_STAT5_SIGNALING	1,163	0,125	0,178	WF
E2F_TARGETS	−2,345	0,000	0,000	CTR
G2M_CHECKPOINT	−2,193	0,000	0,000	CTR
ESTROGEN_RESPONSE_LATE	−1,900	0,000	0,001	CTR
KRAS_SIGNALING_DN	−1,850	0,000	0,001	CTR
PROTEIN_SECRETION	−1,794	0,000	0,001	CTR
OXIDATIVE_PHOSPHORYLATION	−1,743	0,000	0,001	CTR
MITOTIC_SPINDLE	−1,763	0,000	0,002	CTR
MYC_TARGETS_V1	−1,727	0,000	0,002	CTR
BILE_ACID_METABOLISM	−1,690	0,000	0,002	CTR
ADIPOGENESIS	−1,695	0,000	0,002	CTR
PEROXISOME	−1,697	0,002	0,002	CTR
ESTROGEN_RESPONSE_EARLY	−1,702	0,000	0,003	CTR
DNA_REPAIR	−1,608	0,000	0,006	CTR
FATTY_ACID_METABOLISM	−1,610	0,000	0,006	CTR
ANDROGEN_RESPONSE	−1,574	0,002	0,008	CTR
MYOGENESIS	−1,403	0,009	0,035	CTR
P53_PATHWAY	−1,404	0,013	0,037	CTR
CHOLESTEROL_HOMEOSTASIS	−1,411	0,036	0,037	CTR
MTORC1_SIGNALING	−1,280	0,051	0,096	CTR
UV_RESPONSE_DN	−1,260	0,065	0,107	CTR
XENOBIOTIC_METABOLISM	−1,200	0,081	0,159	CTR
APICAL_SURFACE	−1,150	0,249	0,218	CTR
SPERMATOGENESIS	−1,141	0,183	0,219	CTR

Table S3. GSEA analysis of WF + RIBE stimulated vs CTR MDA-MB-468 cell line.

Pathway	Size	NOM *p*-val	FDR *q*-val	FWER *p*-val	Enriched in Population
TNFA_SIGNALING_VIA_NFKB	197	0,000	0,000	0,000	WF+RIBE
MYC_TARGETS_V2	53	0,000	0,001	0,002	WF+RIBE
REACTIVE_OXIGEN_SPECIES_PATHWAY	46	0,000	0,002	0,002	WF+RIBE
INTERFERON_GAMMA_RESPONSE	197	0,000	0,004	0,011	WF+RIBE
ANGIOGENESIS	36	0,004	0,006	0,025	WF+RIBE
ALLOGRAFT_REJECTION	197	0,000	0,007	0,024	WF+RIBE
HYPOXIA	196	0,000	0,009	0,043	WF+RIBE
UV_RESPONSE_UP	151	0,000	0,010	0,057	WF+RIBE
KRAS_SIGNALING_UP	197	0,004	0,012	0,078	WF+RIBE
EPITHELIAL_MESENCHYMAL_TRANSITION	196	0,000	0,016	0,111	WF+RIBE
E2F_TARGETS	195	0,002	0,022	0,177	WF+RIBE
IL6_JAK_STAT3_SIGNALING	84	0,007	0,023	0,169	WF+RIBE
GLYCOLYSIS	197	0,002	0,024	0,202	WF+RIBE
APICAL_JUNCTION	196	0,002	0,031	0,279	WF+RIBE
PANCREAS_BETA_CELLS	40	0,053	0,041	0,376	WF+RIBE
MYC_TARGETS_V1	185	0,017	0,042	0,407	WF+RIBE
UNFOLDED_PROTEIN_RESPONSE	110	0,067	0,076	0,634	WF+RIBE
ESTROGEN_RESPONSE_LATE	196	0,024	0,076	0,678	WF+RIBE
G2M_CHECKPOINT	198	0,028	0,077	0,663	WF+RIBE
TGF_BETA_SIGNALING	53	0,098	0,095	0,776	WF+RIBE
APOPTOSIS	158	0,072	0,100	0,836	WF+RIBE
INFLAMMATORY_RESPONSE	197	0,048	0,100	0,852	WF+RIBE
COAGULATION	135	0,092	0,104	0,836	WF+RIBE
DNA_REPAIR	140	0,066	0,106	0,829	WF+RIBE
WNT_BETA_CATENIN_SIGNALING	42	0,207	0,164	0,951	WF+RIBE
PROTEIN_SECRETION	92	0,000	0,000	0,000	CTR
PEROXISOME	102	0,004	0,025	0,066	CTR
UV_RESPONSE_DN	141	0,004	0,028	0,108	CTR
KRAS_SIGNALING_DN	193	0,004	0,029	0,187	CTR
BILE_ACID_METABOLISM	110	0,004	0,033	0,170	CTR
ANDROGEN_RESPONSE	99	0,015	0,040	0,325	CTR
FATTY_ACID_METABOLISM	153	0,009	0,042	0,375	CTR
CHOLESTEROL_HOMEOSTASIS	71	0,019	0,043	0,307	CTR
ESTROGEN_RESPONSE_EARLY	198	0,069	0,213	0,931	CTR

Table S4. GSEA analysis of RT-WF stimulated vs WF stimulated MDA-MB-468 cell line.

**Pathway**	**NES**	**NOM *p*-val**	**FDR *q*-val**	**Enriched in Population**
E2F_TARGETS	2,884	0,000	0,000	RT-WF
G2M_CHECKPOINT	2,577	0,000	0,000	RT-WF
MYC_TARGETS_V1	2,393	0,000	0,000	RT-WF
MYC_TARGETS_V2	2,027	0,000	0,000	RT-WF
DNA_REPAIR	1,949	0,000	0,000	RT-WF
MITOTIC_SPINDLE	1,936	0,000	0,000	RT-WF
TGF_BETA_SIGNALING	1,497	0,032	0,023	RT-WF
OXIDATIVE_PHOSPHORYLATION	1,416	0,005	0,043	RT-WF
PANCREAS_BETA_CELLS	1,346	0,093	0,071	RT-WF
ESTROGEN_RESPONSE_LATE	1,274	0,060	0,103	RT-WF
EPITHELIAL_MESENCHYMAL_TRANSITION	1,282	0,032	0,104	RT-WF
APICAL_JUNCTION	1,287	0,037	0,109	RT-WF
INTERFERON_ALPHA_RESPONSE	−1,893	0,000	0,003	WF
INTERFERON_GAMMA_RESPONSE	−1,748	0,000	0,005	WF
UNFOLDED_PROTEIN_RESPONSE	−1,779	0,000	0,005	WF
HEME_METABOLISM	−1,786	0,000	0,007	WF
INFLAMMATORY_RESPONSE	−1,587	0,000	0,023	WF
TNFA_SIGNALING_VIA_NFKB	−1,385	0,011	0,136	WF
COMPLEMENT	−1,291	0,038	0,186	WF
XENOBIOTIC_METABOLISM	−1,310	0,040	0,197	WF
HYPOXIA	−1,297	0,046	0,198	WF
NOTCH_SIGNALING	−1,314	0,109	0,217	WF
PEROXISOME	−1,233	0,120	0,228	WF
IL6_JAK_STAT3_SIGNALING	−1,235	0,105	0,244	WF

Table S5. GSEA analysis of WF+RIBE stimulated vs WF stimulated MDA-MB-468 cell line.

Pathway	NES	NOM *p*-val	FDR *q*-val	Enriched in Population
E2F_TARGETS	3,054	0,000	0,000	WF+RIBE
G2M_CHECKPOINT	2,754	0,000	0,000	WF+RIBE
MYC_TARGETS_V1	2,420	0,000	0,000	WF+RIBE
DNA_REPAIR	2,319	0,000	0,000	WF+RIBE
MYC_TARGETS_V2	1,991	0,000	0,000	WF+RIBE
MITOTIC_SPINDLE	1,853	0,000	0,000	WF+RIBE
ESTROGEN_RESPONSE_LATE	1,865	0,000	0,000	WF+RIBE
OXIDATIVE_PHOSPHORYLATION	1,873	0,000	0,000	WF+RIBE
ADIPOGENESIS	1,555	0,000	0,014	WF+RIBE
ESTROGEN_RESPONSE_EARLY	1,446	0,006	0,038	WF+RIBE
MYOGENESIS	1,409	0,008	0,051	WF+RIBE
PANCREAS_BETA_CELLS	1,383	0,068	0,055	WF+RIBE
WNT_BETA_CATENIN_SIGNALING	1,385	0,068	0,058	WF+RIBE
MTORC1_SIGNALING	1,240	0,063	0,172	WF+RIBE
KRAS_SIGNALING_DN	1,212	0,112	0,199	WF+RIBE
FATTY_ACID_METABOLISM	1,177	0,142	0,231	WF+RIBE
REACTIVE_OXIGEN_SPECIES_PATHWAY	1,179	0,225	0,241	WF+RIBE
INTERFERON_ALPHA_RESPONSE	−2,014	0,000	0,000	WF
INTERFERON_GAMMA_RESPONSE	−1,824	0,000	0,002	WF
INFLAMMATORY_RESPONSE	−1,728	0,000	0,004	WF
TNFA_SIGNALING_VIA_NFKB	−1,624	0,000	0,011	WF
COMPLEMENT	−1,587	0,000	0,016	WF
HYPOXIA	−1,501	0,004	0,031	WF
HEME_METABOLISM	−1,467	0,002	0,038	WF
UNFOLDED_PROTEIN_RESPONSE	−1,309	0,063	0,142	WF
UV_RESPONSE_DN	−1,267	0,054	0,185	WF
IL6_JAK_STAT3_SIGNALING	−1,234	0,122	0,213	WF
COAGULATION	−1,218	0,125	0,223	WF
KRAS_SIGNALING_UP	−1,204	0,078	0,227	WF
